# Temporal progression of pupil dilation and gaze behavior to emotion expressions in preschoolers with autism spectrum disorder

**DOI:** 10.1038/s41598-024-58480-2

**Published:** 2024-04-03

**Authors:** Leonie Polzer, Marc Schenk, Naisan Raji, Solvejg Kleber, Christian Lemler, Janina Kitzerow-Cleven, Ziyon Kim, Christine M. Freitag, Nico Bast

**Affiliations:** Department of Child and Adolescent Psychiatry, Psychosomatics and Psychotherapy, Autism Research and Intervention Center of Excellence, University Hospital Frankfurt, Goethe-University, Deutschordenstraße 50, 60528 Frankfurt am Main, Germany

**Keywords:** Autism spectrum disorders, Human behaviour, Eye manifestations, Diagnostic markers, Paediatric research

## Abstract

Previous work has shown divergent pupil dilation (PD) and gaze behavior in individuals with autism spectrum disorder (ASD), which may relate to the development of social difficulties in early life. Here, we investigated temporal dynamics of both phenotypes during naturalistic videos of a person displaying facial emotion expressions in 61 autistic and 61 non-autistic preschoolers. PD was segmented into three serial time components derived from a principal component analysis. Growth curve analysis was applied to analyze changes in looking time on eye and mouth regions over time. Groups did not differ in PD time components. Growth curve analysis revealed initially shorter looking times on the eyes and longer looking times on the mouth in autistic versus non-autistic preschoolers. However, a reversion of this pattern was observed over time, suggesting a delayed compensatory increase in eye attention during prolonged viewing periods in autistic children. Positive and negative associations of PD components and gaze behavior over time indicated a dynamic temporal relationship during emotion viewing. Our findings emphasize the need to apply time-sensitive measures in ecologically valid research, which may index etiological mechanisms of social difficulties in ASD.

## Introduction

Autism Spectrum Disorder (ASD) is a neurodevelopmental condition characterized by difficulties in social interaction and communication as well as repetitive and restricted behaviors^1^. Social communication difficulties in ASD relate to different emotion processing. This has been described as atypical neurophysiological activation^2^ and visual attention to emotion expressions^3^, which may contribute to diverging explicit processing of social information^4^. Aberrations could hence affect efficient behavioral adaptation to others’ emotions in social communication. Additionally, early differences may contribute to cascading effects on social learning^5^. Characterizing neurophysiological activity and visual attention during early development may thus improve the understanding of etiological mechanisms of social-communicative difficulties. Here, we investigated pupil dilation (PD) as a measure of neurophysiological activity and gaze behavior as a measure of visual attention during emotion expression viewing in autistic preschoolers.

PD can be observed as event-related changes in the pupil size during constant lighting conditions. This event-related PD indexes a norepinephrine release of the Locus Coeruleus (LC;^6^). LC activity has been suggested to emphasize neuronal information processing by increasing the signal-to-noise ratio of signal transmission for salient stimuli^7^. Salience describes the level to which stimuli stand out of their environment due to an interplay of bottom-up characteristics (e.g. contrast) and prior knowledge-guided processing (i.e., top-down salience;^8^). Recent research indicated an atypical PD to salience information in ASD^9^. Emotional characteristics contribute to the top-down salience of social stimuli^10^. Thus, we propose atypical salience processing as an underlying mechanism of atypical emotion processing. PD to emotional characteristics could hence inform about neurophysiological salience processing of emotion expressions in ASD.

Atypical neurophysiological activity during face viewing has been suggested by findings of attenuated PD to images^11^ and dynamic videos of faces^12^ in autistic compared to non-autistic preschoolers. ASD symptoms can already be reliably assessed in preschoolers^13^, while processing alterations are unlikely to be mitigated by intervention effects in contrast to older participants. Evidence from the preschool-age might thus be crucial in understanding developmental mechanisms of social-emotional difficulties. Previous studies reported attenuated PD to fear expressions in autistic compared to non-autistic toddlers and preschoolers, however only to unfamiliar but not familiar^14^ and subliminal but not consciously presented stimuli^15^. In older age groups, atypical PD was reported to happy expressions^16,17^, while other studies did not find group difference for PD to specific emotion expressions^18,19^.

Gaze behavior constitutes another determinant of emotion processing. Atypical gaze behavior in ASD has been described as a reduced number of fixations and fixation duration to eyes, which is already observable during early childhood and persists with increasing age^20–22^. In contrast, a meta-analysis reported heterogeneous, but non-significant group differences in gaze behavior to the mouth^21^, which might be dependent on participants’ speech abilities and the involvement of speech cues in the stimulus material^21,23,24^. Findings of a decreased eye fixation time for threatening compared to non-threatening faces in autistic school-aged children^25^ indicated an effect of emotion expressions on gaze behavior differences. However, specific effects may vary across age^3^ and could not be found by a number of other studies^4,17,18^.

Atypical neurophysiological processing and visual attention to faces likely contribute to social-emotional difficulties in autistic individuals. Accordingly, previous studies reported associations between PD to emotion expressions and ASD symptoms^14,15^ as well as gaze behavior relating to poorer emotion recognition performance^4^ and empathy^15^. Further insights into moderating mechanisms of emotion processing could enrich developmental models of social-emotional difficulties in ASD.

Emotion processing is characterized by the involvement of different processing mechanisms during dynamic social situations. The external validity of stimuli and sensitivity of analyses to changes may therefore be crucial for a comprehensive understanding. Dynamic videos compared to static images have been linked to more consistent differences in PD^26^ and visual attention^27^ between autistic and non-autistic participants. The predominant presentation of static images in research may hence be accompanied by an insufficient external validity to uncover processing differences. Moreover, temporal changes of the PD signal and gaze behavior have the potential to reveal patterns that are not detectable after data aggregation. Amongst other methods, the extraction of temporal PD components has been established as a method with increased sensitivity to group differences over conventional measures^28^. The resulting PD components have previously been proposed to reflect diverging processing stages that dominate during successive time intervals. Early components were suggested to reflect sensory processing^29,30^ and alerting^31^. Later components were interpreted as cognitive processing^29^, or orienting and subsequent executive control^30,31^. Extracting time components hence combines the pronounced sensitivity of temporal analyses with an increased interpretability of underlying processes. PD time components could give detailed insights into the differential involvement of processing mechanisms during divergent emotion processing.

Dynamic changes in gaze behavior serve critical functions during social interactions, including efficient information extraction and the modulation of interpersonal contact^32^. Temporal gaze behavior changes have also been investigated by a variety of methods, including scanning path sequences^33^. In an emotion expression paradigm, school-aged autistic compared to non-autistic children showed an differential time course of looking behavior in dependence of the respective emotion expression^25^. This was most consistently characterized by a decreased fixation time on the mouth but not the eyes of happy faces over a 20 s presentation span. Growth curve analyses have been used to directly model time course data, which revealed dynamic specificities of gaze behavior to non-emotional social stimuli that differed between autistic and non-autistic participants^34,35^. Temporal fluctuations during emotion processing may hence be critical to understand difficulties in the fine-tuning of social situations^14^. Still, single value measures are commonly used for PD and gaze behavior investigations. We expect that temporal analyses of responses to dynamic videos would more closely represent emotion processing in naturalistic behavior.

Single studies have investigated the relationship between gaze behavior and PD during the viewing of emotion expressions. Differential links with emotion expressions were suggested by two studies in toddlers and preschoolers. The PD amplitude to fear expressions was related to the number of fixations to the eyes in autistic children^14^ and fixation duration on eyes in both autistic and non-autistic children^15^. This could be explained by shared underlying LC-NE activity that modulates salience processing^9^. While the causality of findings remains unclear, findings suggest a potential association between neurophysiological activity and gaze behavior in visual attention, which might present differentially across processing stages. However, temporal characteristics of this relationship have not been investigated.

Different PD and gaze behavior to faces have been established in ASD, while temporal dynamics of their mutual relationship and dependency on emotion expressions remain unexplored. The current study examines neurophysiological activity and visual attention as assessed by PD and gaze behavior during passive viewing of naturalistic emotion expressions in autistic compared to non-autistic preschoolers. We apply temporal analyses of PD and gaze behavior as sensitive measures of dynamic processes. We expect altered PD and gaze behavior progressions in ASD and investigate how they relate to different emotion expressions. We explore mutual associations of gaze behavior and PD for different emotion expressions. Results may inform about the dynamics of neurophysiological activity and visual attention during naturalistic emotion processing in ASD.

## Methods

### Sample

61 autistic children (ASD group) and 61 non-autistic children (typically developing; TD group) between 18 and 65 months participated in the present study (see Table 1). Groups were matched for developmental age. For autistic participants, the assessment was conducted as part of the baseline measurement of a randomized controlled trial^36^. Participants were recruited from the wait list for therapy at the associated therapy center, local advertisement in kindergartens, social media, and health care institutions. The study was carried out with the approval of the ethics committee of the Department of Medicine at Goethe University Frankfurt (ASD [10/18], TD [361/18]) and in accordance with the Declaration of Helsinki. Caretakers of all participants provided written informed consent for study participation. Trained psychologists confirmed ASD diagnoses according to DSM-5 diagnostic criteria using the German versions of the Autism Diagnostic Observation Schedule 2 (ADOS-2;^37^) and the Autism Diagnostic Interview-Revised (ADI-R;^38^). The ADI-R toddler algorithm^39^ was used for children younger than 4 years.

**Table 1:** Sample description.

	ASD (*n* = 61)	TD (*n* = 61)	comparison	*p*-value
Sex (male/female)	51/10	32/29	χ^2^ = 12.21, *df* = 1	< 0.001
DA (in months)	29.78 (11.77)	33.19 (10.75)	*t* = 1.67, *df* = 119.03	0.098
Age (in months)	48.03 (10.02)	32.84 (10.82)	*t* = 8.04, *df* = 119.29	< 0.001
IQ/DQ^a^	60.84 (18.63)	101.76 (11.98)	*t* = −14.42, *df* = 102.37	< 0.001
ADOS-2 CSS	7.07 (1.58)	–	–	–
ADI-R total score	40.48 (8.71)	–	–	–
ADI-R toddler score	18.97 (3.52)	–	–	–
SRS-16 sum score	27.96 (7.7)	4.60 (2.81)	*t* = 20.99, *df* = 67.03	< 0.001
CBCL 1 ½–5 Int T-score	64.11 (8.67)	46.14 (9.16)	*t* = 10.57, *df* = 107.96	< 0.001
CBCL 1 ½–5 Ext T-score	59.87 (9.34)	46.74 (7.67)	*t* = 8.02, *df* = 100.39	< 0.001
CBCL 1 ½–5 Total T-score	62.15 (9.02)	44.39 (7.33)	*t* = 11.29, *df* = 100.33	< 0.001
Onscreen time (%)	80.37 (9.02)	83.9 (8.29)	*t* = −2.20, *df* = 113.40	0.029

Values are presented as mean (SD) except where otherwise specified. Onscreen time is defined as the total time spent looking at the screen.

ADI-R, Autism diagnostic interview revised; ADOS-2 CSS, Autism diagnostic observation schedule 2 calibrated severity score; ASD, autism spectrum disorder; CBCL, Child behavior checklist; DA/TA, developmental age/test age; Ext, externalizing; Int, internalizing; IQ/DQ, intelligence quotient/developmental quotient; SRS, Social responsiveness scale; TD, typically developing children.

^a^Depending on age and test performance, cognitive ability measures were either assessed with the WPPSI-III (IQ) or Bayley-III (DQ).

Exclusion criteria were a nonverbal IQ/developmental quotient < 30 and a developmental age < 12 months as assessed by the German versions of either the Bayley Scales of Infant Development—Third Edition (Bayley-III;^40^) or the Wechsler Preschool and Primary Scale of Intelligence-III (WPPSI-III;^41^) depending on participants’ age and understanding of instructions (see^12^ for further information). Further exclusion criteria were severe sensory impairments, cerebral palsy, chronic neurological disorder, unstable epilepsy, neurodegenerative disorder, Rett/Angelman Syndrome, severe psychosocial deprivation, attachment disorder, institutional upbringing as well as parents not being fluent in German. Within the TD group, participants with one or more scales of the German version of the Child Behavior Checklist 1 ½–5^42^ in clinical ranges (T > 65) or a T-score > 75 in the German version of the Social Responsiveness Scale (SRS;^43^) were excluded. The SRS T-Score was interpolated from the sum score of the Social Responsiveness Scale—16 item version (SRS-16), which is highly associated with the SRS long form (r = 0.98;^44^).

Our group previously published findings on a luminance adaptation and a social attention paradigm in an overlapping sample^12^.

### Apparatus

Eye-tracking measurements were done while participants either were seated on a highchair or on the caregiver’s lap. Participants could move their head freely within 50–80 cm screen distance, while the stimulus material was presented on a presentation screen of 1920 × 1080 pixels. Eye-tracking data was assessed by the Tobii TX300 eye-tracker at a sampling rate of 300 Hz. Before the experiment was started, a 5-point calibration was performed.

### Stimuli

**Figure 1:**
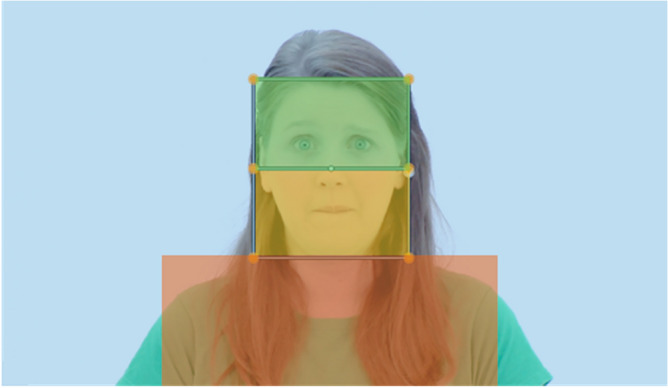
Illustration of the Area of Interest (AOI) definition on one frame of a stimulus. The eye region (green) and mouth region (yellow) were matched in size. Body (red) and background (blue) were not included in the analyses due to sparse observations. Fixed AOI sizes were used across stimuli. Stimuli were derived from a validated stimulus set of emotion expressions^45^.

The task consisted of 12 videos with durations between 4.6 and 12.0 s. Due to varying video lengths, we analyzed the first 4.5 s of each video. Videos were derived from a validated set of naturalistic emotional stimuli that are freely available for a scientific use^45^. We only selected videos that showed a high validity rating and were rated as high intensity in the original data set (mean correct identification: 88%, range: 73–97%). Each video displayed one of four actors (man, woman, boy, girl) in front of a white background presenting one of three emotion expressions (fear, happy, neutral). Only the face and upper torso were visible, and no additional gestures were presented (see Fig. 1). Each video started out with an actor’s direct gaze into the camera, which stayed mostly directed into the camera over the whole video duration. In videos displaying a fear expression, the actors occasionally turned their gaze away from the camera. The stimuli were presented in blocks of three videos. The order of appearance was randomized. Before each video, a blinking fixation cross (2.5 Hz for 2 s) was presented in the center of the screen. The four resulting blocks appeared at different time points during a comprehensive eye-tracking battery. The eye-tracking battery was coded in Psychtoolbox-3 for MATLAB and can be retrieved online: https://github.com/nicobast/BOSCA_battery. The total duration of the eye-tracking assessment was about 25 min.

### Preprocessing

Trials with less than 50% of available data after preprocessing (see below) were excluded (ASD: total = 13.3% of all trials, *M* = 3.22, *SD* = 2.38 number of excluded trials; TD: total = 6.1% of all trials, *M* = 1.48, *SD* = 1.80; *t*(111.76) = 4.58, *p* < 0.001, Cohen’s *d* = 0.83). Data points were excluded if the gaze was not located on the screen.

#### Pupil dilation

Data and code are available from the corresponding author. Data preprocessing was based on recent recommendations^46^. Pupil data were preprocessed with the exclusion of invalid pupil sizes (< 2 mm and > 8 mm), dilation speed outliers (> median dilation speed + 3*median absolute deviation [MAD]), and data points 25 ms before and after data gaps likely representing blinks (> 75 ms and ≤ 250 ms). A two-pass approach was applied, in which 1) data points that deviated more than 3 MAD from each participant’s individual trend line and 2) data points that deviated more than 3 MAD from the resulting trend line were excluded. Interpolated values that considered the individual offset between eyes were used to replace missing values of one eye. Pupil response was defined as the mean pupil size across both eyes. Missing data for ≤ 300 ms were interpolated. To further handle noise, a moving average algorithm (30 ms span) was applied. The pupil response was normalized by subtracting the mean pupil size during the first 500 ms of each trial, respectively. There were no differences in baseline pupil size between groups (*F*(1,107.53) = 1.35, *p* = 0.248). The baseline size measurements (first 500 ms) were excluded for analyses.

#### Gaze behavior

The gaze position was averaged across both eyes. If data was missing for one eye, gaze positions were interpolated from the other eye while considering the individual offset between eyes. A velocity-based fixation algorithm was applied with a data-driven velocity threshold to identify saccades^47^. Data was denoised with a Savitsky-Golay filter (for coefficients, see http://www.statistics4u.info/fundstat_eng/cc_savgol_coeff.html). Fixations with durations < 100 ms and > 2500 ms were excluded. Data for which no fixation was identified were removed from fixation analyses. Fixation locations were computed as the mean value of gaze positions during the respective fixation.

Areas of interest (AOI) were defined frame-wise for each video using Blender^48^. We defined two AOI: eye region, and mouth region (see Fig. 1). As all actors were displayed in similar size in each video, AOI sizes were held constant across frames and stimuli. Eye regions included moving muscle areas under the eyes (lower bound above the tip of the nose). Mouth regions included the nasolabial folds. Eye and mouth regions were matched in size (23% of x-axis, 23% of y-axis) and aligned on the x-axis. Initially defined background and body regions were not included in analyses since there were no fixations on these regions in the majority of trials (background: total = 77% of trials, ASD = 73%, TD = 79%; body: total = 79%, ASD = 65%, TD = 87%; eyes: total = 16%, ASD = 23%, TD = 12%; mouth: total = 37%, ASD = 33%, TD = 39%). Groups did not differ in the mean number of excluded fixations across trials (*t*(111.46) = 1.44, *p* = 0.15).

In total, three outcomes were investigated as measures of differential gaze behavior to AOI between groups: (1) number of fixations, (2) fixation duration (defined as mean across fixations), and (3) looking time progression within trials. The number of fixations and the fixation duration were derived from the fixation algorithm. Both measures have been used as standard measures in similar paradigms^21^. Looking time progression was defined as the change of gaze behavior over time to index temporal dynamics of gaze behavior. For this, we calculated looking time as the individual sum of gaze samples within 50 ms windows for each AOI in each trial. We chose a narrower time window compared to previous studies^25,35^ to increase the resolution of dynamic patterns during shorter viewing times. Broadening time windows to 100 ms and 200 ms did not change the overall pattern of results (see Fig. S2 in the supplementary material).

### Data analysis

All analyses were done using R^49^ version 4.0.3 with the additional packages ggplot2^50^, lme4^51^, lmerTest^52^, emmeans^53^, psych^54^. We used linear mixed effect models (LMMs) to investigate pupil dilation, gaze behavior and their association. For all models, we included age, sex and onscreen time (total time spent looking at the screen) as covariates as well as participant as random intercept. In PD models, gaze deviation from the center of the screen was included as additional covariate to control for effects of different gaze behavior. For all models, continuous variables were scaled and centered. Tukey-tests were used for post-hoc testing. We report significant effect sizes as marginalized fixed effects (β) with 95% confidence intervals. Full models can be found in the supplements.

#### Pupil dilation

**Figure 2:**
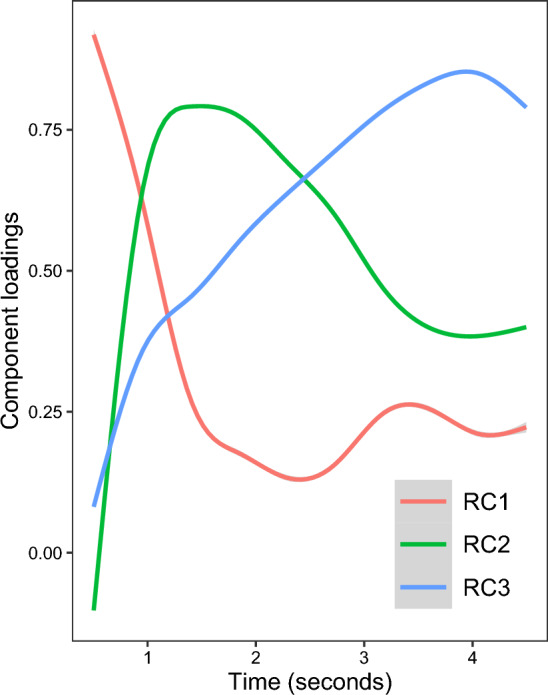
Pupil dilation loadings on rotated components (RC) over time. For each RC, one pupil dilation measure was extracted from the time windows of the highest loading time points (T-RC): T-RC1: 500–920 ms, T-RC2: 923–2470 ms, T-RC3: 2473–4500 ms.

A principal component analysis (PCA) was applied to identify time components of the pupil response. Three components were extracted based on visual inspection of the scree plot (see Table S1 and Fig. S1 in the supplementary material). The components were varimax-rotated, which resulted in three rotated components (RCs) representing different underlying temporal clusters for pupil response during trials (explained variance: RC1 = 14%, RC2 = 38%, RC3 = 48%; see Fig. 2). For each RC, we defined one time window (T-RC) based on the highest loading time points (T-RC1: 500–920 ms, T-RC2: 923–2470 ms, T–RC3: 2473–4500 ms). Subsequently, PD was calculated as the mean pupil response during each time window. An LMM was applied to examine group differences in PD over time. PD during all T-RCs was included as dependent variable. The interactions between group (ASD, TD), emotion expression (happy, fear, neutral) and time windows (T-RC1, T-RC2, T-RC3) were analyzed as fixed effects.

#### Gaze behavior

Group differences in gaze behavior depending on emotion expression and AOI (gaze on eyes vs. mouth region) were assessed by two LMMs with (1) the number of fixations and (2) fixation duration as dependent variable. The interactions between group, AOI and emotion expression were included as fixed effects.

Looking time progression in dependence of emotion expression and AOI was analyzed with a growth curve analysis. For this, the looking time (see above) was included as dependent variable in an LMM. We included a polynomial fixed effect of time to model the temporal dynamics of the looking time progression. A fourth-degree polynomial provided the best increment in fit, while considering parsimony (see Table S5 in the supplementary material for comparisons of polynomial fits). The interactions between group, AOI, expression and time were included as fixed effects. Post-hoc comparisons were calculated for every 250 ms.

#### Association of pupil dilation and gaze behavior

Three LMMs were used to analyze the effects of PD during each time window (T-RC1, T-RC2, T-RC3) on the number of fixations. Three further LMMs were used to analyze the effect on fixation duration. The interactions between PD, the expression and AOI were included as fixed effects.

The dynamic association between PD and looking time progression was analyzed by LMMs for each time window. One model for each AOI per time window was calculated to avoid overfitting. The mean looking time within the respective time spans of the time windows were included as dependent variable. PD and the interaction with emotion expression were included as fixed effects.

## Results

### Pupil dilation

**Figure 3:**
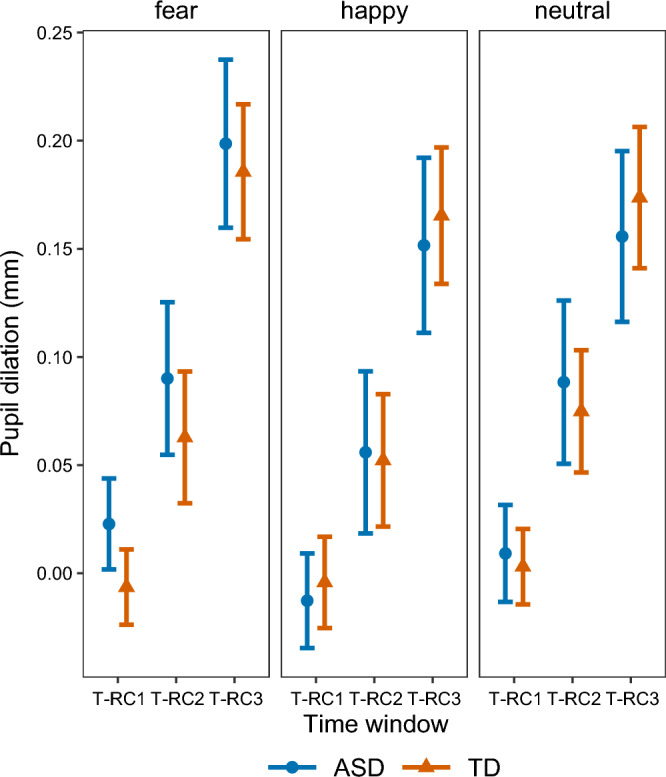
Mean pupil dilation per time window (T-RC). Error bars represent standard errors. PD increased over time but did not differ between emotion expression.

We analyzed whether PD was predicted by time window and its interaction with group and emotion expression. There was a significant main effect of time window on PD (*F*(2,2459.75) = 52.54, *p* < 0.001). PD increased between the subsequent time windows (T-RC1 vs. T-RC2: β =  − 0.19, 95% CI: − 0.298 to − 0.087, T-RC1 vs. T-RC3: β =  − 0.47, 95% CI: − 0.572 to − 0.358, T-RC2 vs. T-RC3: β =  − 0.27, 95% CI: − 0.379 to − 0.167, see Fig. 3), which indicated an PD increase within trials. There were no significant interactions between group, emotion expression and time window (see supplementary Table S2).

### Gaze behavior

#### Number of fixations

**Figure 4:**
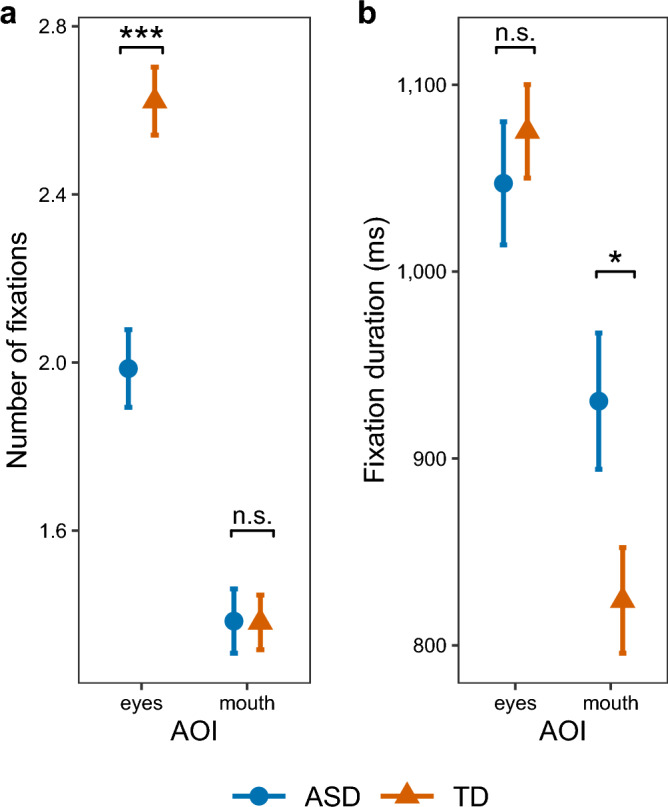
Gaze behavior for eye and mouth regions. Error bars represent standard errors. (**a**) Mean number of fixations: ASD participants showed a smaller number of fixations on the eye region. (**b**) Mean fixation duration: ASD participants showed a longer fixation duration of the mouth region. Significance levels: *p* < 0.05*, *p* < 0.01**, *p* < 0.001***.

The number of fixations was explained by an interaction between AOI and group (*F*(1,1739.64) = 15.171, *p* < 0.001). Autistic compared to non-autistic participants presented a lower number of fixations on the eye region (β =  − 0.38, 95% CI: − 0.551 to − 0.205), but not on the mouth region (β = 0.032, 95% CI: − 0.141 to 0.205, see Fig. 4a). In addition, there was a significant interaction effect of AOI and expression (*F*(2,1739.64) = 6.83, *p* = 0.001). Post-hoc comparisons showed a higher number of fixations on the mouth for happy compared to neutral expressions (β = 0.23, 95% CI: 0.046 to 0.407) and for happy compared to fear expressions (β = 0.18, 95% CI: 0.036 to 0.005). There was neither a significant interaction between group and expression nor between group, AOI and expression (see supplementary Table S3).

#### Fixation duration

Fixation duration was explained by an interaction between AOI and group (*F*(1,1345.02) = 6.29, *p* = 0.012). Post-hoc comparisons revealed a longer fixation duration on the mouth region in autistic compared to non-autistic participants (β = 0.24, 95% CI: 0.024 to 0.457; see Fig. 4b). There were no significant differences between groups for fixation duration on the eye region (β =  − 0.03, 95% CI: − 0.236 to 0.169). We did not find significant interaction effects with expression (see supplementary Table S4).

#### Looking time progression

**Figure 5:**
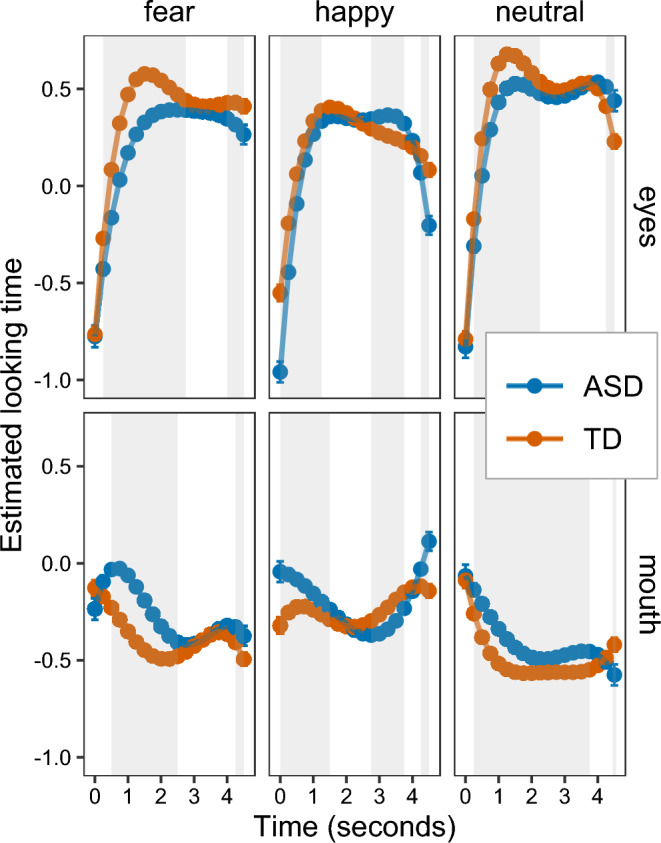
Estimated marginal means of looking times within trials based on a linear mixed model. Error bars represent standard errors. Between-group differences in looking time was tested for every 250 ms. Grey background indicates time points with significant between-group differences (*p* < 0.05).

Looking time was explained by significant four-wise interactions between group, expression, AOI and a linear effect of time (*F*(2,145546) = 30.33, *p* < 0.001), a quadratic effect of time (*F*(2,145546) = 25.04, *p* < 0.001), a cubic effect of time (*F*(2,145546) = 23.14, *p* < 0.001), and a quartic effect of time (*F*(2,145546) = 23.41, *p* < 0.001). These represented differently shaped time courses of gaze behavior for autistic compared to non-autistic participants in dependence of emotion expressions and AOI (see Fig. 5). While there was an initial increase in looking time to the eyes for all emotion expressions across groups, looking time to the mouth was predominantly characterized by an overall decrease during the first half of the stimulus presentation. Post-hoc comparisons were used to disentangle diverging patterns of looking time progressions between groups.

For the eye region, autistic participants showed an initially shorter looking time compared to non-autistic participants across expressions (e.g. at 1000 ms, fear: β =  − 0.30, 95% CI: − 0.355 to − 0.246; happy: β =  − 0.07, 95% CI: − 0.121 to − 0.014; neutral: β =  − 0.20, 95% CI: − 0.255 to − 0.142). During later stages of trials, autistic participants showed time points of longer looking time to happy (e.g. at 3500 ms: β = 0.12, 95% CI: 0.060 to 0.171) and neutral expressions (e.g. at 4500 ms: β = 0.21, 95% CI: 0.082 to 0.338).

For the mouth region, autistic compared to non-autistic participants showed initially longer looking times (e.g. at 1000 ms, fear: β = 0.29, 95% CI: 0.234 to 0.344; happy: β = 0.08, 95% CI: 0.030 to 0.137; neutral: β = 0.18, 95% CI: 0.121 to 0.234). Only for happy expressions, there was a relatively extended sequence with shorter looking time for autistic participants (e.g. at 3250 ms, β =  − 0.11, 95% CI: − 0.164 to − 0.061). Only at the end of the trials, autistic participants showed shorter looking times on the mouth region of neutral expressions (at 4500 ms, β =  − 0.16, 95% CI: − 0.283 to − 0.028).

### Association of pupil dilation and gaze behavior

#### Number of fixations

The number of fixations was neither explained by PD during T-RC1, T-RC2, T-RC3 nor their interaction with expression and AOI (see supplementary Tables S7, S8 and S9).

#### Fixation duration

Fixation duration was explained by a three-wise interaction between PD during T-RC1, AOI and expression (*F*(2,1130.70) = 4.60, *p* = 0.010). For fixation duration on the mouth region, PD during T-RC1 showed different trends for happy vs. neutral expressions (β =  − 1.41, 95% CI: − 2.474 to − 0.340), with a larger PD being associated with a higher fixation duration on neutral expressions (β = 1.13, 95% CI: 0.442 to 1.181), but not on happy expressions (β =  − 0.28, 95% CI: − 0.846 to 0.291).

PD during T-RC2 showed an interaction with AOI (*F*(1,1152.76) = 6.97, *p* = 0.008). A larger PD was associated with a shorter fixation duration on the eye region (β =  − 0.19, 95% CI: − 0.370 to − 0.004), but not on the mouth region (β = 0.19, 95% CI: − 0.03 to 0.41). Neither PD during T-RC3 nor its interaction with expression and AOI explained fixation duration (see supplementary Table S12).

#### Looking time progression

A higher looking time on the mouth (*F*(1,544.97) = 5.91, *p* = 0.015) but not the eye region (*F*(1,690.83) = 1.95, *p* = 0.163) was explained by a smaller PD during T-RC1. PD during T-RC2 was neither associated with the looking time on the eye or mouth region. A smaller PD during T-RC3 was associated with increased looking time on the eye region (*F*(1,764.01) = 4.26, *p* = 0.039), as well as on the mouth region (*F*(1,685.27) = 5.25, *p* = 0.022). There were no interactions with emotion expression (see Tables S13–S18).

## Discussion

Autistic individuals have been reported to show an atypical pupil dilation (PD) and gaze behavior to faces. Here, we investigated PD and gaze behavior as well as their mutual association in response to naturalistic emotion expressions. To increase sensitivity and investigate time-dependent mechanisms, temporal progression analyses were applied to PD and gaze behavior. Within social contexts, these temporal processes may affect efficient emotion recognition^4^, which is relevant for social adaptation. In etiological models, they have been suggested to contribute to the emergence of social difficulties in early development^5^. While we did not find evidence for atypical PD, autistic compared to non-autistic preschoolers showed differential gaze behavior patterns in dependence of facial region, emotion expression and, notably, outcome measure (i.e. fixation number, fixation duration, looking time). Differential relationships between PD and gaze behavior supported dynamic interdependencies during emotion processing.

PD measures were extracted from three principal components and reflected PD during sequential time windows. Following previous interpretations of PD time components^29,30^, we propose sensory processing to underlie the early component RC1, orienting being reflected in RC2, and higher-order cognitive processing underlying the late component RC3. Across groups, PD was smallest during the beginning of trials (T-RC1) and increased over time (for T-RC2 and T-RC3, respectively). Contrary to our hypothesis, we did not find PD group differences. This may suggest similar neurophysiological activity during emotion expression viewing in autistic and non-autistic preschoolers. Similar PD contradicts earlier findings of group differences to social stimuli as reported for single-value measures^11^ and time progression analyses^19^. An atypical salience perception in ASD has been proposed before^9^, which could explain differing findings by an interaction between social salience and ASD diagnosis. Assuming a dimensional construct of social salience, naturalistic emotion expressions with a direct gaze may trigger neurophysiological activation as an alertness mechanism. Compared to non-emotional or static stimuli, naturalistic emotional material might therefore carry an inherent biological salience and induce similar increases in pupil size across groups. Conflicting this assumption, naturalistic emotion expressions compared to static stimuli have previously been reported to lead to especially sensitive PD group differences in 3–16 year olds^26^, which might be due to age differences between studies. In line with other studies, we did not find differential PD progressions between groups for different emotion expressions^18,19^. Other studies reported between-group differences in PD single-value measures^15^ and temporal progression for specific emotions^16^. Due to considerable variations in study designs, previous findings are difficult to integrate into a comprehensive narrative. The specific influence of sample characteristics, stimulus material and analysis strategies are necessary to be addressed in future studies.

As expected, we found significant group differences in gaze behavior. Notably, group differences showed divergent patterns between gaze behavior measures. Autistic compared to non-autistic participants displayed a smaller number of fixations on the eye region, but a longer fixation duration on the mouth region. Temporal progression analyses revealed an initially lower looking time for the eye region in autistic compared to non-autistic children, but a higher looking time for the mouth region. These findings correspond to an established line of research pointing to atypical gaze behavior to eyes^20,22^. While previous findings on gaze behavior to the mouth are heterogeneous^21^, our results suggest that sensitive outcome measures are required to quantify atypical mouth attention. However, study differences may scale with differing language skills. For children during speech and language acquisition phases, increased gaze behavior to the mouth was proposed as a mechanism of redundant multi-sensory input to facilitate speech perception^21,23^. Prevalence rates of delayed speech in autistic children are high^55^, which emphasizes the need to further identify non-redundant contributions to atypical gaze behavior.

Moreover, the looking time progression revealed differential patterns for emotion expressions between ASD and TD groups over time. In dependence of the emotion expression, the initial looking pattern—of a shorter looking time on eyes but longer looking time on the mouth in autistic preschoolers—was partly reversed over time. Especially happy expressions elevated prolonged periods with longer looking time on the eyes but shorter looking time on the mouth for autistic compared to non-autistic participants during the second half of the trial. Differences between emotion expressions may relate to findings suggesting a varying informativity of eye and mouth regions for the recognition of different emotions^3^. Interestingly, happy expressions have previously been reported to lead to increased mouth attention in non-autistic participants, which might explain less pronounced initial group differences to the eye region and more consistent later periods of increased mouth attention in the TD group^56^. In contrast, fear and neutral expressions are proposed to typically attract more attention to the eye region^56^. The temporal characteristics of our findings contradict results of a previous study investigating temporal gaze behavior during longer emotion expression videos in school-aged children^25^. The authors reported an increased mouth fixation time in non-autistic compared to autistic participants, which was relatively stable over time in response to happy expressions and occasionally observable in response to fear expressions. These differences might be explained by a lacking control of total gaze time, which resulted in TD showing generally increased looking times. Nonetheless, our and previous findings^34,35^ indicate atypical temporal dynamics of gaze behavior in ASD. Atypical attention to the eyes has previously been proposed to have potential impacts on social difficulties including emotion expression recognition^4^. Diverging temporal processing may play a critical role in this relationship by altering social information extraction^32^ and delaying emotion expression recognition. Our findings suggest that autistic compared to non-autistic preschoolers may compensate for initially decreased attention to eyes only after given sufficient time to process emotion expressions. This would contradict previous suggestions of general increased mouth attention as a strategy to compensate for difficulties with extracting information from the eyes^57^. Future research may unveil how temporal aspects of emotion expression recognition relate to difficulties in the fine tuning of social interactions.

In exploratory analyses, PD and gaze behavior were associated differentially across time, AOI and emotion expressions. A larger pupil dilation during high-loading time points of the first principal component (T-RC1) was associated with a shorter looking time on the mouth. PD during T-RC1 most likely reflects sensory processing including a pupillary light adaptation in the beginning of trials. Due to lower looking times on the mouth region in non-autistic participants, the mouth might initially not be highly relevant for information extraction from emotion expressions. Attention to the mouth might not induce as much cognitively driven pupillary dilation antagonizing the light-induced pupillary constriction. Interestingly, a larger PD during T-RC1 was also associated with a higher fixation duration on the mouth region of neutral expressions. In contrast to looking time, fixation duration was defined across trials, which might explain diverging directions of associations. A larger PD during T-RC2 was associated with a shorter fixation duration on the eye region across trials. This suggests an association of an increased neurophysiological activity during orienting with less pervasive attention on eyes. This could be driven by a faster re-orienting between fixations during increased neurophysiological activity. Moreover, a smaller PD during T-RC3 was associated with increased looking time on the eye and the mouth region. Exhaustive information extraction towards the end of trials may hence be accompanied by cognitive downregulation of neurophysiological activity across groups. This may reflect a general change from a neurophysiological mode that fosters stimulus exploitation back to an exploration mode, which renders the organism sensitive for new incoming stimuli^58^. Due to the exploratory nature of these analyses, results need to be interpreted cautiously and require replication. However, alongside with findings of associations between PD and fixation behavior to eyes and mouth regions by previous studies^14,15^, our findings suggest that further research is required to characterize the temporal interplay of neurophysiological activity and gaze behavior in emotion processing.

Limitations of the current study include group differences in sample characteristics. Age has been reported to influence gaze behavior^59^ as well as PD^60^. We controlled for age and sex differences in statistical models. Moreover, groups were matched for developmental age but differed in IQ. This corresponds to increased prevalences of intellectual impairment in the ASD population^61^ and is unlikely to impact implicit emotion processing. Visual attention has however been linked to language development^24^ and culture^20^, which we did not assess in the current sample. Future studies are invited to include additional control groups. In fear expression stimuli, the actors’ gaze was not consistently directed into the camera. While one previous study reported differential PD between autistic compared to non-autistic school-aged children for direct but not averted gaze^17^, a study in preschoolers did not find group differences in gaze behavior or PD for direct vs. averted gaze^62^. A systematic influence on our results is unlikely due to the only occasionally averted gaze and no distinct abnormalities in results for fear expressions compared to other emotion expressions. Additionally, PD estimates have been shown to be influenced by gaze behavior due to the rotation of the eye ball^63^, which is inevitable for assessing naturalistic gaze patterns. While influences of gaze behavior cannot be ruled out, we accommodated for this effect by controlling for gaze deviation in PD analyses.

Taken together, our results support similar neurophysiological activity but visual attention differences to emotion expressions in autistic compared to non-autistic preschoolers. Differential relationships of pupil dilation components with visual attention demonstrate a potential interplay of mechanisms, while underpinning the advantages to differentiate underlying processing stages. We show that visual attention in autistic compared to non-autistic preschoolers seems to differ not only in spatial but also temporal dimensions, which might translate to difficulties in the fine tuning of real-word social interactions. Our findings emphasize the utility of temporal progression analyses on emotion processing to increase the ecological validity of future research.

### Supplementary Information

Supplementary Information.

## Data Availability

The datasets generated during and/or analysed during the current study are available from the corresponding author on reasonable request.
