# Supplementary material for: Temporal progression of pupil dilation and gaze behavior to emotion expressions in preschoolers with autism spectrum disorder
Source: Sci Rep. 2024 Apr 3;14:7843. doi: 10.1038/s41598-024-58480-2 (PMC10991397; doi:10.1038/s41598-024-58480-2)
Supplement: Supplementary file 1 — Supplementary Information. [file 41598_2024_58480_MOESM1_ESM.pdf]

Supplementary information

Temporal progression of pupil dilation and gaze behavior to emotional expressions in preschoolers with autism spectrum disorder

Leonie Polzer, Marc Schenk, Naisan Raji, Solvejg Kleber, Christian Lemler, Janina Kitzerow-Cleven, Ziyon Kim, Christine M. Freitag, Nico Bast

Content

1 Pupil dilation 2
2 Gaze behavior 4
3 Association of PD and gaze behavior 11
  3.1 Number of fixations 11
  3.2 Fixation duration 14
  3.3 Looking time progression 17

1 Pupil dilation

Table S 1. Comparisons of (non-rotated) principal components of pupil dilation over time.

	PC1	PC2	PC3	PC4	PC5	PC6	PC7	PC8	PC9
SS loadings	955.71	75.55	48.39	28.44	17.56	12.77	9.74	6.18	5.07
Proportion Var	0.80	0.06	0.04	0.02	0.02	0.01	0.01	0.01	0.00
Cumulative Var	0.80	0.86	0.90	0.92	0.94	0.95	0.96	0.96	0.97
Proportion Explained	0.82	0.07	0.04	0.03	0.02	0.01	0.01	0.01	0.00
Cumulative Proportion	0.82	0.89	0.93	0.96	0.97	0.98	0.99	1.00	1.00

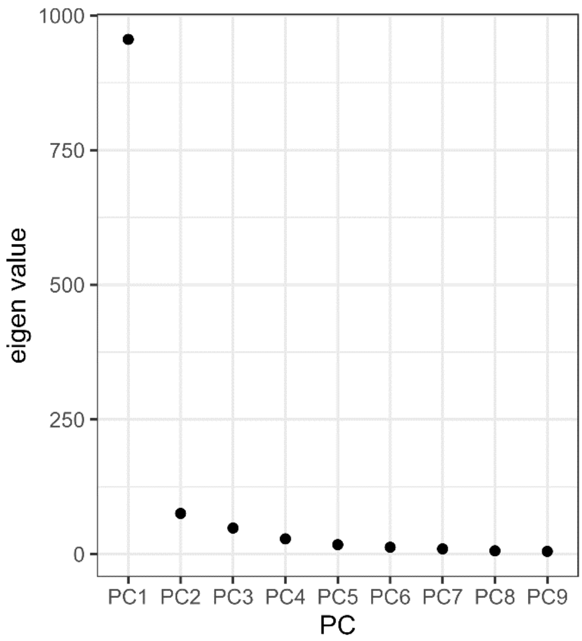

Figure S 1. Scree-plot of (non-rotated) principal components of pupil dilation over time.

Table S 2. Linear mixed model predicting PD.

**Fixed effects**	**β**	**SE**	**Sum Sq**	**Mean Sq**	**Num DF**	**DenDF**	**F value**	**p**
group (TD)	-0.07	0.15	0.01	0.01	1	97.08	0.01	.930
expression			2.40	1.20	2	2542.80	1.43	.239
-happy	-0.11	0.12						
-neutral	-0.02	0.13						
time window			87.50	43.75	2	2459.75	52.54	.000
-T-RC2	0.20	0.12						
-T-RC3	0.50	0.12						
center deviation	0.00	0.22	0.00	0.00	1	2373.84	0.00	.973
age	0.05	0.05	1.02	1.02	1	98.22	1.23	.271
sex (female)	-0.05	0.05	0.30	0.30	1	96.90	0.36	.550
onscreen time	0.13	0.02	28.90	28.90	1	2445.21	34.71	.000
group x expression			1.70	0.85	2	2543.24	1.02	.360
-TD x happy	0.16	0.16						
-TD x neutral	0.09	0.16						
group x time window			0.24	0.12	2	2456.13	0.14	.868
-TD x T-RC2	0.00	0.16						
-TD x T-RC3	0.04	0.16						
expression x time window			0.72	0.18	4	2454.20	0.22	.929
-happy x T-RC2	-0.01	0.17						
-neutral x T-RC2	0.02	0.18						
-happy x T-RC3	-0.05	0.17						
-neutral x T-RC3	-0.08	0.18						
group x expression x time window			0.08	0.02	4	2454.33	0.02	.999
-TD x happy x T-RC2	-0.03	0.22						
-TD x neutral x T-RC2	-0.03	0.22						
-TD x happy x T-RC3	-0.04	0.22						
-TD x neutral x T-RC3	0.01	0.22						
**Random effects**	**Var**	**Std**						
participant	0.14	0.38						
residual	0.83	0.91						

2 Gaze behavior

Table S 3. Linear mixed model predicting the number of fixations.

**Fixed effects**	**β**	**SE**	**Sum Sq**	**Mean Sq**	**Num DF**	**DenDF**	**F value**	**p**
group (TD)	0.43	0.13	6.33	6.33	1	105.70	7.41	.008
AOI (mouth)	-0.38	0.12	117.11	117.11	1	1739.64	137.28	.000
expression			1.23	0.61	2	1818.13	0.72	.487
-*happy*	-0.00	0.12						
-*neutral*	0.15	0.13						
age	0.08	0.03	4.60	4.60	1	103.42	5.39	.022
sex (female)	0.15	0.07	4.70	4.69	1	100.67	5.50	.021
onscreen time	0.05	0.02	4.43	4.43	1	1511.85	5.20	.023
group x AOI			12.94	12.94	1	1739.64	15.17	.000
-*TD x mouth*	-0.34	0.16						
group x expression			0.69	0.35	2	1817.76	0.40	.668
-*TD x happy*	-0.10	0.15						
-*TD x neutral*	-0.07	0.16						
AOI x expression			11.67	5.83	2	1739.64	6.84	.001
-*mouth x happy*	0.22	0.17						
-*mouth x neutral*	-0.13	0.18						
group x AOI x expression			0.15	0.08	2	1739.64	0.09	.916
-*TD x mouth x happy*	0.04	0.22						
-*TD x mouth x neutral*	-0.05	0.22						
**Random effects**	**Var**	**Std**						
participant	0.04	020						
residual	0.85	0.92						

Table S 4. Linear mixed model predicting the fixation duration.

Fixed effects	β	SE	Sum Sq	Mean Sq	Num DF	DenDF	F value	p
group (TD)	0.18	0.15	1.14	1.14	1	99.47	1.29	.259
AOI (mouth)	-0.12	0.16	32.16	32.16	1	1345.72	36.38	.000
expression			0.13	0.07	2	1321.15	0.08	.926
-happy	0.07	0.14						
-neutral	0.05	0.15						
age	-0.06	0.04	1.69	1.69	1	95.94	1.91	.170
sex (female)	-0.17	0.08	4.16	4.16	1	90.15	4.71	.033
onscreen time	0.14	0.03	24.10	24.10	1	1178.57	27.26	.000
group x AOI			5.56	5.56	1	1345.02	6.29	.012
-TD x mouth	-0.50	0.19						
group x expression			0.25	0.12	2	1321.55	0.14	.870
-TD x happy	-0.29	0.18						
-TD x neutral	-0.14	0.18						
AOI x expression			0.66	0.33	2	1284.71	0.37	.690
-mouth x happy	-0.17	0.21						
-mouth x neutral	-0.04	0.22						
group x AOI x expression			4.35	2.18	2	1284.44	2.47	.086
-TD x mouth x happy	0.55	0.26						
-TD x mouth x neutral	0.14	0.27						
**Random effects**	**Var**	**Std**						
participant	0.06	0.24						
residual	0.88	0.94						

Table S 5. Model comparisons of polynomial fits for prediction of looking time progression.

	AIC	BIC	Chisq	Pr(>Chisq)	AIC.decrement	BIC.decrement
m1	391185.0	391471.8	NA	NA	0.000	0.000
m2	388565.9	388971.4	2643.15	0	-2619.15	-2500.48
m3	387446.5	387970.6	1143.41	0	-1119.41	-1000.73
m4	386929.9	387572.7	540.56	0	-516.56	-397.89
m5	386520.8	387282.2	433.16	0	-409.16	-290.49
m6	386093.5	386973.6	451.27	0	-427.27	-308.60

Table S 6. Linear mixed model predicting looking time.

Fixed effects	β	SE	Sum Sq	Mean Sq	Num DF	DenDF	F value	p
time	1.97	0.19	666.48	666.48	1	145672.33	801.16	.000
time^2	-4.73	0.80	432.50	432.50	1	145679.12	519.90	.000
time^3	4.57	1.15	305.12	305.12	1	145682.19	366.78	.000
time^4	-1.64	0.54	231.62	231.62	1	145682.80	278.43	.000
group (TD)	0.14	0.02	0.00	0.00	1	93.33	0.00	.964
expression			3.83	1.91	2	106323.87	2.30	.100
-happy	-0.03	0.01						
-neutral	0.15	0.01						
AOI (mouth)	-0.53	0.01	15126.31	15126.31	1	145546.14	18183.00	.000
onscreen time	0.05	0.00	242.16	242.16	1	16160.56	291.09	.000
age	-0.00	0.01	0.27	0.27	1	91.94	0.33	.568
sex (female)	-0.01	0.01	0.30	0.29	1	90.57	0.35	.554
time x group	0.90	0.24	1.10	1.10	1	145670.27	1.32	.251
time^2 x group	-3.36	0.99	0.77	0.77	1	145678.49	0.93	.335
time^3 x group	3.69	1.41	0.65	0.65	1	145682.13	0.78	.376
time^4 x group	-1.27	0.66	0.58	0.58	1	145682.91	0.70	.405
time x expression			0.19	0.10	2	145665.01	0.12	.891
-time x happy	1.07	0.26						
-time x neutral	1.07	0.28						
time^2 x expression			0.01	0.01	2	145659.64	0.01	.994
-time^2 x happy	-4.94	1.11						
-time^2 x neutral	-4.43	1.17						
time^3 x expression			0.06	0.03	2	145659.14	0.04	.964
-time^3 x happy	7.29	1.60						
-time^3 x neutral	5.71	1.68						
time^4 x expression			0.14	0.07	2	145659.42	0.08	.920
-time^4 x happy	-3.51	0.75						
-time^4 x neutral	-2.35	0.79						

Fixed effects	β	SE	Sum Sq	Mean Sq	Num DF	DenDF	F value	p
group x expression			2.97	1.49	2	106048.87	1.79	.168
-TD x happy	-0.12	0.02						
-TD x neutral	-0.08	0.02						
time x AOI	-1.04	0.27	698.38	698.38	1	145545.81	839.51	.000
time^2 x AOI	-0.05	1.14	263.59	263.59	1	145545.76	316.86	.000
time^3 x AOI	1.98	1.63	131.13	131.13	1	145545.76	157.63	.000
time^4 x AOI	-1.14	0.76	83.62	83.62	1	145545.77	100.51	.000
group x AOI	-0.27	0.02	207.14	207.14	1	145546.14	248.99	.000
expression x AOI			1030.94	515.47	2	145546.14	619.63	.000
-happy x mouth	0.08	0.02						
-neutral x mouth	-0.30	0.02						
time x group x expression			0.74	0.37	2	145665.70	0.45	.640
-time x TD x happy	-1.85	0.33						
-time x TD x neutral	-0.26	0.34						
time^2 x group x expression			0.67	0.33	2	145660.26	0.40	.670
-time^2 x TD x happy	6.99	1.39						
-time^2 x TD x neutral	0.36	1.43						
time^3 x group x expression			0.60	0.30	2	145659.72	0.36	.698
-time^3 x TD x happy	-9.14	1.99						
-time^3 x TD x neutral	0.49	2.06						
time^4 x group x expression			0.56	0.28	2	145659.98	0.34	.713
-time^4 x TD x happy	4.01	0.93						

Fixed effects	β	SE	Sum Sq	Mean Sq	Num DF	DenDF	F value	p
-time^4 x TD x neutral	-0.65	0.96						
time x group x AOI	-2.01	0.33	8.48	8.48	1	145545.93	10.19	.001
time^2 x group x AOI	7.11	1.40	8.41	8.41	1	145545.89	10.12	.001
time^3 x group x AOI	-7.56	2.00	4.26	4.26	1	145545.88	5.12	.024
time^4 x group x AOI	2.53	0.94	1.60	1.60	1	145545.88	1.92	.166
time x expression x AOI			65.71	32.85	2	145546.03	39.49	.000
-time x happy x mouth	-2.04	0.37						
-time x neutral x mouth	-2.35	.039						
time^2 x expression x AOI			60.49	30.24	2	145546.04	36.35	.000
-time^2 x happy x mouth	9.10	1.57						
-time^2 x neutral x mouth	9.00	1.65						
time^3 x expression x AOI			52.90	26.45	2	145546.05	31.80	.000
-time^3 x happy x mouth	-13.13	2.26						
-time^3 x neutral x mouth	-10.92	2.38						
time^4 x expression x AOI			46.88	23.44	2	145546.07	28.17	.000
-time^4 x happy x mouth	6.28	1.06						

Fixed effects	β	SE	Sum Sq	Mean Sq	Num DF	DenDF	F value	p
-time^4 x neutral x mouth	4.28	1.06						
group x expression x AOI			69.57	34.79	2	145546.14	41.81	.000
-TD x happy x mouth	0.22	0.02						
-TD x neutral x mouth	0.10	0.03						
time x group x expression x AOI			50.47	25.23	2	145545.67	30.33	.000
-time x TD x happy x mouth	3.46	0.47						
-time x TD x neutral x mouth	0.71	0.48						
time^2 x group x expression x AOI			41.66	20.83	2	145545.60	25.04	.000
-time^2 x TD x happy x mouth	-12.56	1.97						
-time^2 x TD x neutral x mouth	-0.97	2.03						
time^3 x group x expression x AOI			38.50	19.25	2	145545.60	23.14	.000
-time^3 x TD x happy x mouth	16.05	2.82						
-time^3 x TD x neutral x mouth	-1.34	2.91						
time^4 x group x expression x AOI			38.95	19.47	2	145545.61	23.41	.000

Fixed effects	β	SE	Sum Sq	Mean Sq	Num DF	DenDF	F value	p
-time^4 x TD x happy x mouth	-6.96	1.32						
-time^4 x TD x neutral x mouth	1.67	1.36						
**Random effects**	**Var**	**Std**						
participant	0.00	0.05						
residual	0.83	0.91						

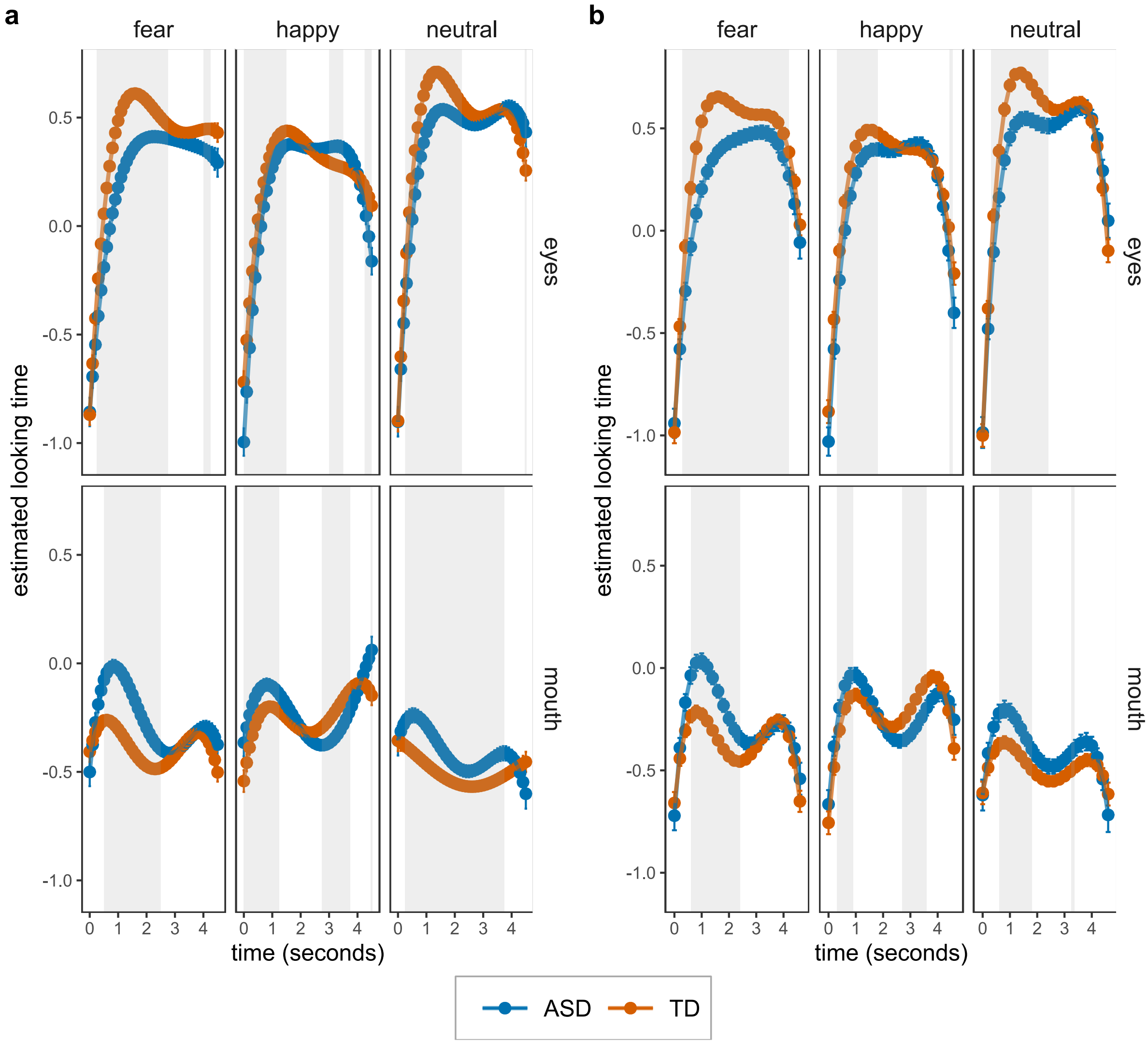

Figure S 2. Estimated marginal means of looking times within trials based on two additional linear mixed models. Figures show results based on looking time as calculated by the sum of gaze samples within sequential a) 100 ms time windows and b) 200 ms time windows. Error bars represent standard errors. Between-group differences in looking time was tested for every 250 ms. Grey background indicates time points with significant between-group differences (p<.05).

3 Association of PD and gaze behavior

3.1 Number of fixations

Table S 7. Linear mixed model predicting number of fixations by PD during T-RC1.

**Fixed effects**	**β**	**SE**	**Sum Sq**	**Mean Sq**	**Num DF**	**DenDF**	**F value**	**p**
PD	-0.05	0.06	0.74	0.74	1	1568.46	0.85	.356
expression			0.99	0.50	2	1573.26	0.57	.565
-happy	-0.08	0.08						
-neutral	0.15	0.08						
AOI (mouth)	-0.57	0.08	127.25	127.25	1	1498.20	146.68	.000
age	0.02	0.03	0.36	0.36	1	111.15	0.41	.522
sex (female)	0.25	0.07	12.34	12.34	1	98.08	14.23	.000
onscreen time	0.04	0.03	2.02	2.02	1	1235.03	2.32	.128
PD x expression			0.21	0.11	2	1587.35	0.12	.885
-PD x happy	0.03	0.08						
-PD x neutral	0.13	0.09						
PD x AOI	0.12	0.08	0.53	0.53	1	1498.20	0.61	.434
expression x AOI			18.60	9.30	2	1498.20	10.72	.000
-happy x mouth	0.28	0.11						
-neutral x mouth	-0.24	0.12						
PD x expression x AOI			2.66	1.33	2	1498.20	1.53	.216
-PD x happy x mouth	-0.05	0.11						
-PD x neutral x mouth	-0.20	0.12						
**Random effects**	**Var**	**Std**						
participant	0.05	0.22						
residual	0.87	0.93						

Table S 8. Linear mixed model predicting number of fixations by PD during T-RC2.

**Fixed effects**	**β**	**SE**	**Sum Sq**	**Mean Sq**	**Num DF**	**DenDF**	**F value**	**p**
PD	0.01	0.06	2.64	2.64	1	1483.46	3.05	.081
expression			0.84	0.42	2	1589.24	0.49	.615
-happy	-0.08	0.08						
-neutral	0.14	0.08						
AOI (mouth)	-0.57	0.08	128.87	128.87	1	1513.24	148.77	.000
age	0.02	0.03	0.27	0.27	1	109.97	0.31	.576
sex (female)	0.25	0.07	12.88	12.88	1	96.81	14.87	.000
onscreen time	0.04	0.03	1.57	1.57	1	1241.31	1.81	.179
PD x expression			0.69	0.325	2	1613.20	0.40	.671
-PD x happy	-0.00	0.08						
-PD x neutral	0.08	0.08						
PD x AOI	0.01	0.08	0.13	0.13	1	1513.24	0.14	.704
expression x AOI			19.06	9.53	2	1513.24	11.00	.000
-happy x mouth	0.27	0.11						
-neutral x mouth	-0.25	0.11						
PD x expression x AOI			2.25	1.13	2	1513.24	1.30	.273
-PD x happy x mouth	0.10	0.11						
-PD x neutral x mouth	-0.08	0.12						
**Random effects**	**Var**	**Std**						
participant	0.05	0.21						
residual	0.87	0.93						

Table S 9. Linear mixed model predicting number of fixations by PD during T-RC3.

**Fixed effects**	**β**	**SE**	**Sum Sq**	**Mean Sq**	**Num DF**	**DenDF**	**F value**	**p**
PD	-0.00	0.06	2.13	2.13	1	1515.38	2.46	.117
expression			0.81	0.40	2	1586.63	0.47	.627
-happy	-0.08	0.08						
-neutral	0.15	0.08						
AOI (mouth)	-0.57	0.08	131.24	131.24	1	1509.68	151.73	.000
age	0.02	0.03	0.29	0.29	1	109.49	0.33	.566
sex (female)	0.25	0.07	12.79	12.79	1	97.18	14.78	.000
onscreen time	0.04	0.03	1.94	1.94	1	1243.44	2.24	.135
PD x expression			0.65	0.32	2	1610.49	0.37	.688
-PD x happy	-0.01	0.08						
-PD x neutral	0.07	0.08						
PD x AOI	0.04	0.08	0.74	0.74	1	1509.68	0.85	.356
expression x AOI			18.27	9.14	2	1509.68	10.56	.000
-happy x mouth	0.26	0.11						
-happy x neutral	-0.25	0.11						
PD x expression x AOI			3.31	1.66	2	1509.68	1.92	.148
-PD x happy x mouth	0.11	0.11						
-PD x neutral x mouth	-0.11	0.12						
**Random effects**	**Var**	**Std**						
Participant	0.05	0.21						
residual	0.86	0.93						

3.2 Fixation duration

Table S 10. Linear mixed model predicting fixation duration by PD during T-RC1.

**Fixed effects**	**β**	**SE**	**Sum Sq**	**Mean Sq**	**Num DF**	**DenDF**	**F value**	**p**
PD	0.04	0.06	0.59	0.59	1	1170.56	0.69	.408
expression			0.31	0.16	2	1154.27	0.18	.835
-happy	-0.13	0.09						
-neutral	-0.07	0.09						
AOI (mouth)	-0.41	0.10	28.46	28.46	1	1172.54	33.04	.000
age	-0.04	0.04	0.84	0.84	1	103.15	0.97	.326
sex (female)	-0.16	0.08	4.05	4.05	1	89.32	4.70	.033
onscreen time	0.17	0.03	25.49	25.49	1	985.94	29.60	.000
PD x expression			3.75	1.88	2	1185.80	2.18	.114
-PD x happy	-0.09	0.08						
-PD x neutral	-0.15	0.09						
PD x AOI	0.00	0.10	3.61	3.61	1	1124.41	4.20	.041
expression x AOI			1.65	0.82	2	1119.10	0.96	.385
-happy x mouth	0.18	0.13						
-neutral x mouth	0.11	0.14						
PD x expression x AOI			7.92	3.96	2	1130.70	4.60	.010
-PD x happy x mouth	-0.02	0.13						
-PD x neutral x mouth	0.36	0.14						
**Random effects**	**Var**	**Std**						
participant	0.06	0.24						
residual	0.86	0.93						

Table S 11. Linear mixed model predicting fixation duration by PD during T-RC2.

**Fixed effects**	**β**	**SE**	**Sum Sq**	**Mean Sq**	**Num DF**	**DenDF**	**F value**	**p**
PD	-0.04	0.06	0.00	0.00	1	1122.00	0.00	.993
expression			0.18	0.09	2	1167.75	0.11	.899
-happy	-0.12	0.09						
-neutral	-0.06	0.09						
AOI (mouth)	-0.41	0.10	29.54	29.54	1	1185.34	34.00	.000
age	-0.03	0.04	0.74	0.74	1	103.20	0.86	.357
sex (female)	-0.17	0.07	4.83	4.83	1	88.91	5.55	.021
onscreen time	0.18	0.03	27.70	27.70	1	979.67	31.88	.000
PD x expression			1.39	0.70	2	1198.62	0.80	.449
-PD x happy	-0.03	0.09						
-PD x neutral	-0.07	0.09						
PD x AOI	0.06	0.10	6.05	6.05	1	1152.76	6.97	.008
expression x AOI			1.69	0.85	2	1131.69	0.97	.378
-happy x mouth	0.18	0.13						
-neutral x mouth	0.08	0.14						
PD x expression x AOI			3.66	1.83	2	1139.74	2.11	.122
-PD x happy x mouth	0.01	0.13						
-PD x neutral x mouth	0.25	0.14						
**Random effects**	**Var**	**Std**						
participant	0.05	0.22						
residual	0.87	0.93						

Table S 12. Linear mixed model predicting fixation duration by PD during T-RC3.

Fixed effects	β	SE	Sum Sq	Mean Sq	Num DF	DenDF	F value	p
PD	-0.04	0.06	1.72	1.72	1	1149.46	1.97	.161
expression			0.22	0.11	2	1167.64	0.12	.883
-happy	-0.12	0.09						
-neutral	-0.06	0.09						
AOI (mouth)	-0.41	0.10	28.75	28.75	1	1183.35	32.82	.000
age	-0.03	0.04	0.81	0.81	1	102.56	0.93	.338
sex (female)	-0.18	0.07	5.18	5.18	1	89.63	5.91	.017
onscreen time	0.18	0.03	28.75	28.75	1	965.22	32.82	.000
PD x expression			1.14	0.57	2	1195.68	0.65	.522
-PD x happy	-0.06	0.09						
-PD x neutral	-0.04	0.09						
PD x AOI	-0.04	0.10	0.88	0.88	1	1151.95	1.01	.316
expression x AOI			1.54	0.77	2	1130.94	0.88	.414
-happy x mouth	0.18	0.13						
-neutral x mouth	0.09	0.14						
PD x expression x AOI			1.92	0.96	2	1143.45	1.09	.336
-PD x happy x mouth	0.09	0.14						
-PD x neutral x mouth	0.21	0.14						
**Random effects**	**Var**	**Std**						
participant	0.05	0.22						
residual	0.88	0.94						

3.3 Looking time progression

Table S 13. Linear mixed model predicting looking time on the eye region by PD during T-RC1.

**Fixed effects**	**β**	**SE**	**Sum Sq**	**Mean Sq**	**Num DF**	**DenDF**	**F value**	**p**
PD	0.03	0.06	1.33	1.33	1	690.83	1.96	.163
expression			2.26	1.13	2	626.53	1.66	.191
-happy	0.02	0.08						
-neutral	0.13	0.08						
onscreen time	0.26	0.04	36.60	36.60	1	703.56	53.90	.000
age	-0.03	0.06	0.21	0.21	1	98.25	0.31	.577
sex (female)	0.02	0.12	0.02	0.02	1	88.39	0.03	.858
PD x expression			0.90	0.45	2	655.08	0.66	.516
-PD x happy	-0.02	0.08						
-PD x neutral	0.06	0.08						
**Random effects**	**Var**	**Std**						
participant	0.25	0.50						
residual	0.68	0.82						

Table S 14. Linear mixed model predicting looking time on the mouth region by PD during T-RC1.

**Fixed effects**	**β**	**SE**	**Sum Sq**	**Mean Sq**	**Num DF**	**DenDF**	**F value**	**p**
PD	-0.02	0.09	4.22	4.22	1	544.97	5.92	.015
expression			1.52	0.76	2	507.51	1.06	.346
-happy	-0.05	0.09						
-neutral	-0.13	0.09						
onscreen time	0.06	0.04	1.46	1.43	1	571.75	2.02	.153
age	0.05	0.06	0.52	0.52	1	112.03	0.73	.394
sex (female)	-0.10	0.14	0.34	0.34	1	102.56	0.48	.489
PD x expression			1.55	0.77	2	536.64	1.08	.339
-PD x happy	-0.08	0.09						
-PD x neutral	-0.14	0.10						
**Random effects**	**Var**	**Std**						
participant	0.30	0.54						
residual	0.71	0.84						

Table S 15. Linear mixed model predicting looking time on the eye region by PD during T-RC2.

Fixed effects	β	SE	Sum Sq	Mean Sq	Num DF	DenDF	F value	p
PD	0.00	0.05	0.72	0.72	1	760.51	1.35	.246
expression			14.32	7.16	2	719.06	13.38	.000
-happy	-0.26	0.06						
-neutral	0.04	0.06						
onscreen time	0.27	0.03	38.54	38.54	1	793.24	72.02	.000
age	-0.17	0.06	3.97	3.97	1	105.41	7.42	.008
sex (female)	0.04	0.13	0.05	0.05	1	97.89	0.10	.751
PD x expression			0.50	0.25	2	733.50	0.47	.627
-PD x happy	-0.05	0.06						
-PD x neutral	-0.06	0.07						
**Random effects**	**Var**	**Std**						
participant	0.34	0.59						
residual	0.54	0.73						

Table S 16. Linear mixed model predicting looking time on the mouth region by PD during T-RC2.

Fixed effects	β	SE	Sum Sq	Mean Sq	Num DF	DenDF	F value	p
PD	-0.07	0.06	1.77	1.77	1	634.33	3.07	.080
expression			23.59	11.80	2	594.12	20.49	.000
-happy	0.023	0.07						
-neutral	-0.25	0.08						
onscreen time	0.12	0.04	6.01	6.01	1	662.39	10.43	.001
age	0.03	0.07	0.12	0.12	1	103.80	0.21	.649
sex (female)	-0.03	0.15	0.02	0.02	1	98.69	0.04	.841
PD x expression			0.22	0.11	2	603.65	0.19	.824
-PD x happy	0.04	0.07						
-PD x neutral	-0.00	0.08						
**Random effects**	**Var**	**Std**						
participant	0.42	0.64						
residual	0.58	0.76						

Table S 17. Linear mixed model predicting looking time on the eye region by PD during T-RC3.

Fixed effects	β	SE	Sum Sq	Mean Sq	Num DF	DenDF	F value	p
PD	-0.03	0.05	2.49	2.49	1	764.01	4.26	.039
expression			7.71	3.85	2	715.72	6.61	.001
-happy	-0.22	0.07						
-neutral	-0.01	0.07						
onscreen time	0.42	0.03	94.39	94.39	1	792.96	161.90	.000
age	-0.06	0.05	0.69	0.69	1	102.69	1.19	.278
sex (female)	0.02	0.11	0.01	0.01	1	96.27	0.02	.880
PD x expression			0.31	0.16	2	736.51	0.27	.766
-PD x happy	-0.04	0.07						
-PD x neutral	-0.04	0.07						
**Random effects**	**Var**	**Std**						
participant	0.23	0.48						
residual	0.58	0.76						

Table S 18. Linear mixed model predicting looking time on the mouth region by PD during T-RC3.

Fixed effects	β	SE	Sum Sq	Mean Sq	Num DF	DenDF	F value	p
PD	-0.08	0.05	3.10	3.10	1	685.27	5.25	.022
expression			23.87	11.94	2	638.60	20.18	.000
-happy	0.19	0.07						
-neutral	-0.27	0.07						
onscreen time	0.23	0.04	25.73	25.73	1	715.60	43.50	.000
age	-0.01	0.06	0.02	0.02	1	103.72	0.03	.866
sex (female)	0.07	0.13	0.16	0.16	1	97.55	0.27	.607
PD x expression			0.34	0.17	2	658.01	0.29	.748
-PD x happy	-0.02	0.07						
-PD x neutral	0.04	0.08						
**Random effects**	**Var**	**Std**						
participant	0.33	0.57						
residual	0.59	0.77						
